# Supplementary material for: Conformation and Domain Movement Analysis of Human Matrix Metalloproteinase-2: Role of Associated Zn2+ and Ca2+ Ions
Source: Int J Mol Sci. 2019 Aug 27;20(17):4194. doi: 10.3390/ijms20174194 (PMC6747341; doi:10.3390/ijms20174194)
Supplement: Supplementary file 1 [file ijms-20-04194-s001.pdf]

Supporting Information for: Conformation and Domain Movement Analysis of Human Matrix Metalloproteinase-2: role of associated Zn^{2+} and Ca^{2+} ions.

Leah Voit-Ostricki[1], Sándor Lovas[2], and Charles R. Watts[1,3,*]

[1] Department of Neurosurgery, Mayo Clinic Health System-Franciscan Healthcare in La Crosse, Wisconsin, 54601, USA; voitost.leah@gmail.com

[2] Department of Biomedical Sciences, Creighton University, Omaha, Nebraska, 68178, USA; SandorLovas@creighton.edu

[3] Department of Neurologic Surgery, Mayo Clinic, Rochester, Minnesota, 55905, USA; charles.watts@parknicollet.com

* Correspondence: charles.watts@parknicollet.com; Tel.: +1-952-993-3200, Fax: +1-952-993-7407

Received: date; Accepted: date; Published: date

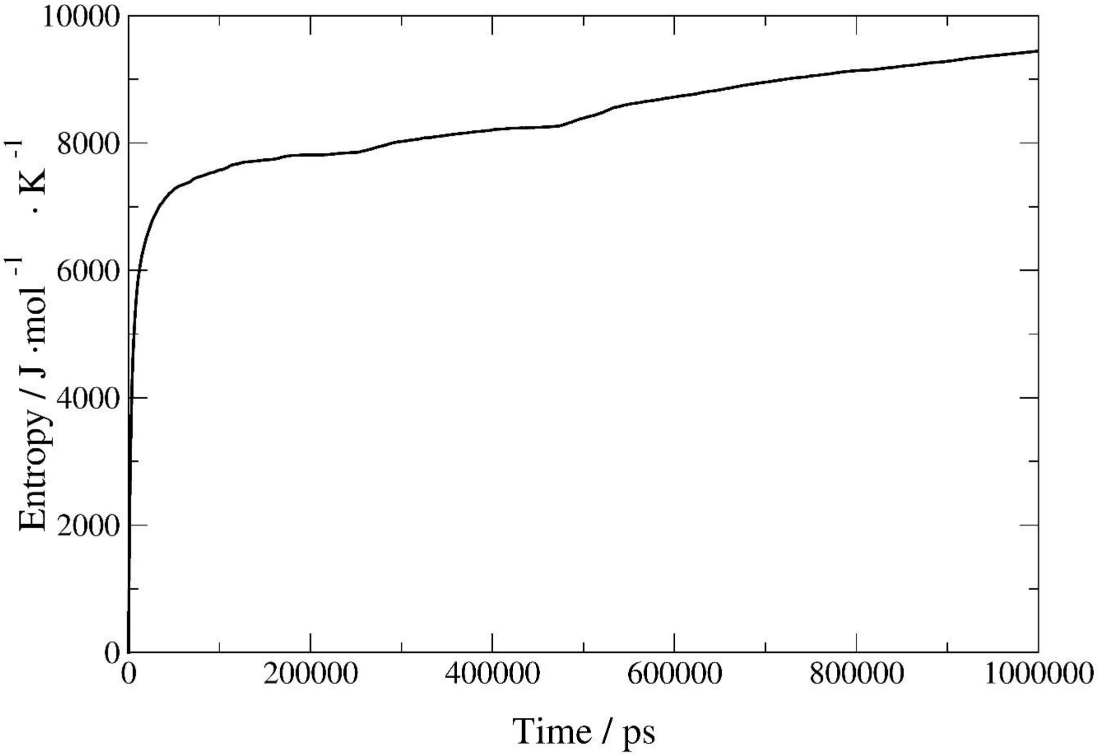

Figure S1. Configurational entropy of MMP-2 (calculated from the Cα-trace, Zn^{2+} and Ca^{2+} coordinates) as a function of time, demonstrating the 200000 ps necessary for system equilibration.

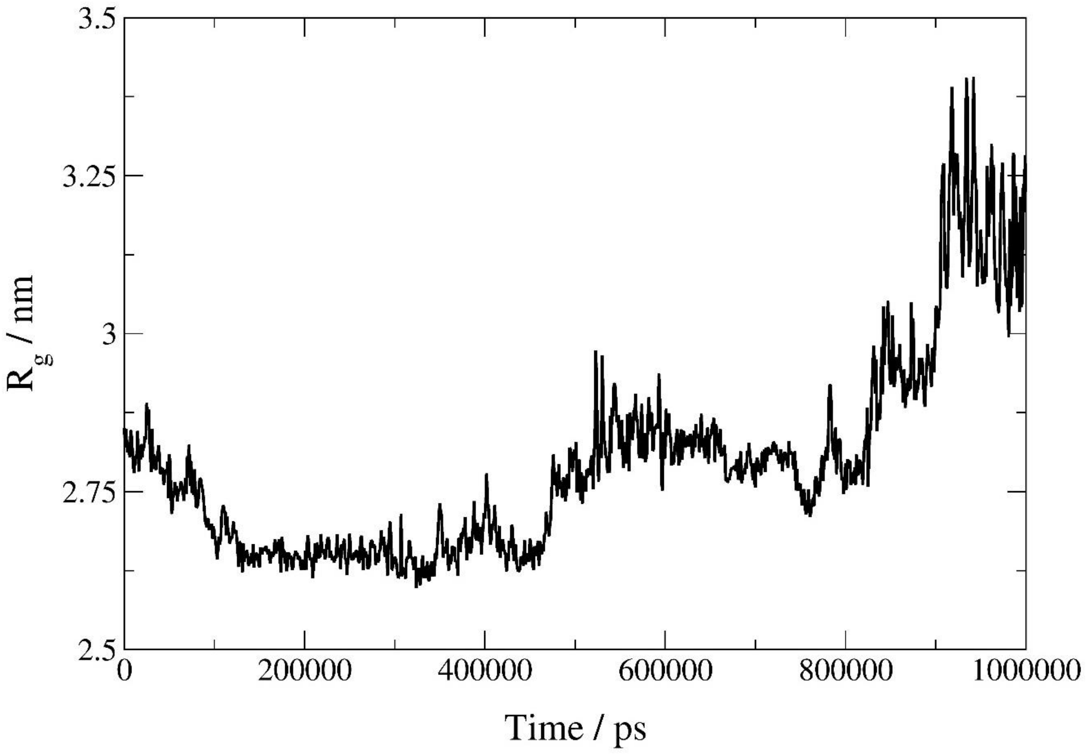

Figure S2. The radius of gyration (R_g) as a function of time.

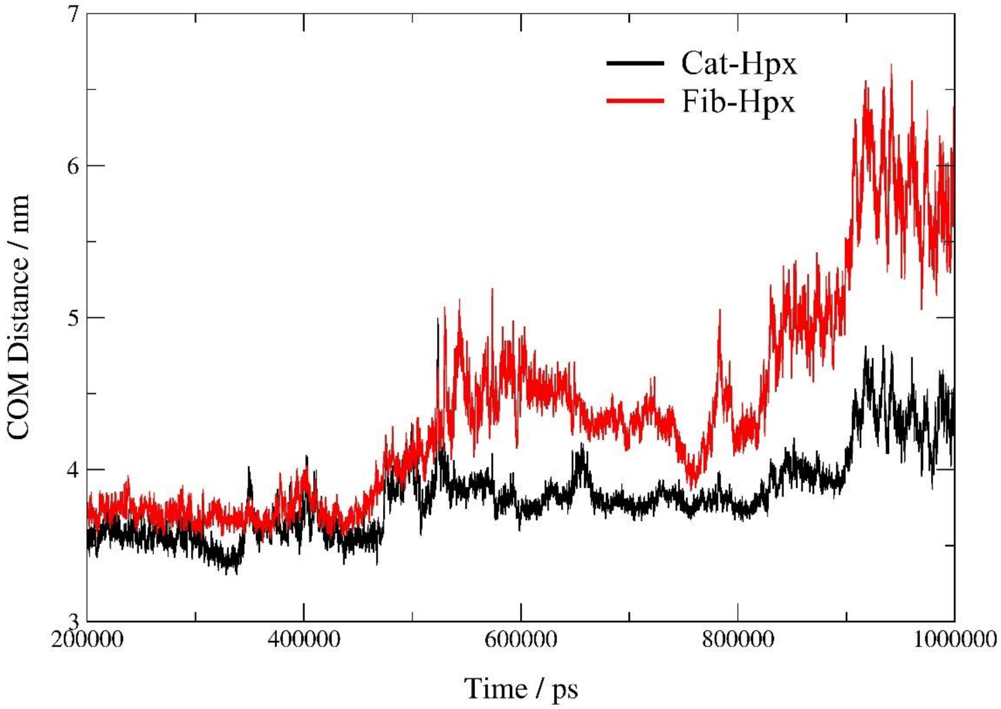

Figure S3. The Center of mass (COM) distance as a function of time between the Cat and Hpx (black) and Fib and Hpx (red) domains, respectively.

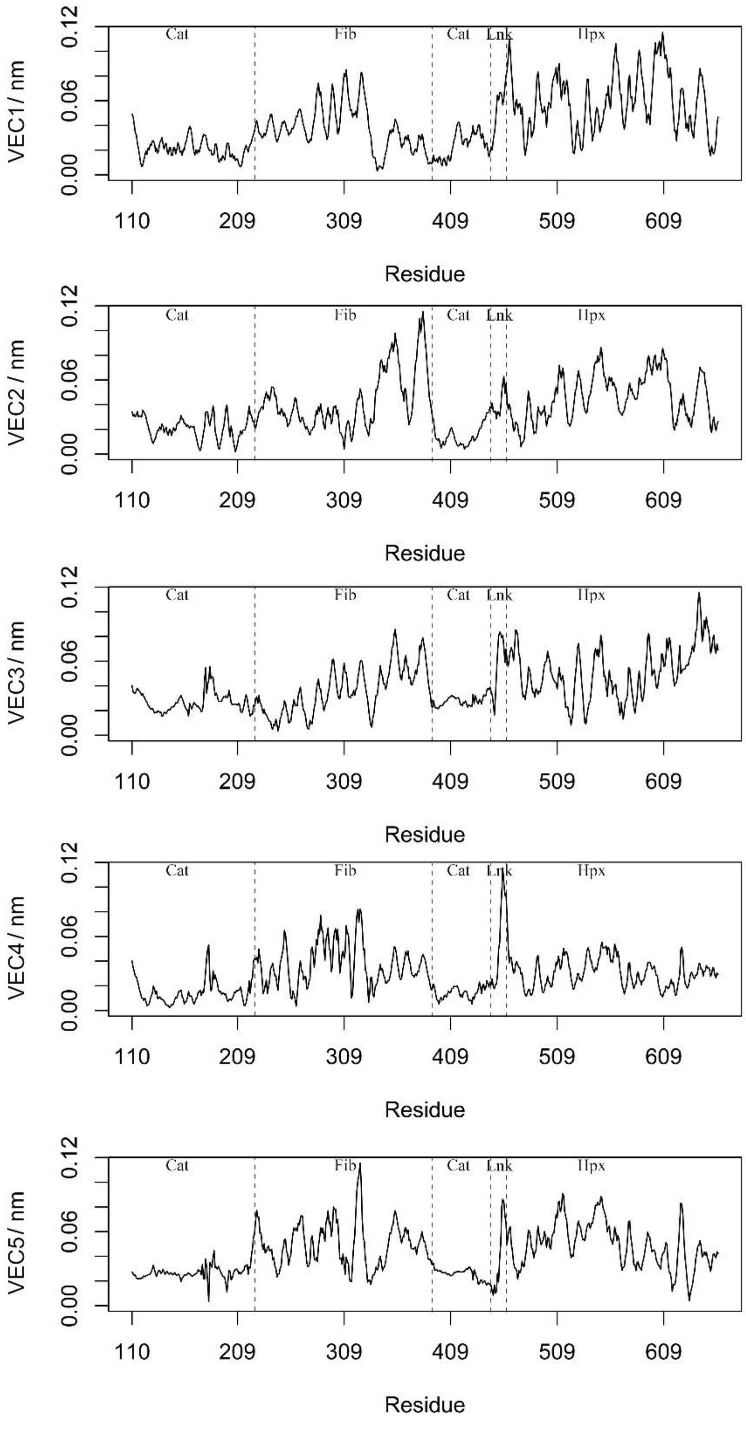

Figure S4. Cα-trace RMSF plots of the top 5 eigenvectors.
